# Integration of Coke and CNMs with Bitumen: Synthesis, Methods, and Characterization

**DOI:** 10.3390/nano15110842

**Published:** 2025-05-31

**Authors:** Muhammad Hashami, Yerdos Ongarbayev, Yerbol Tileuberdi, Yerzhan Imanbayev, Ainur Zhambolova, Aliya Kenzhegaliyeva, Zulkhair Mansurov

**Affiliations:** 1 Faculty of Chemistry and Chemical Technology, Al-Farabi Kazakh National University, 71, Al-Farabi Ave., Almaty 050040, Kazakhstan; mg.hashami2010@gmail.com (M.H.); zmansurov@kaznu.edu.kz (Z.M.); 2 Department of Chemistry, Faculty of Education, Institute of Higher Education Mirwais Khan Nika Zabul, Qalat 4001, Afghanistan; 3 Institute of Combustion Problems, 172, Bogenbai Batyr Str., Almaty 050012, Kazakhstan; er.tileuberdi@gmail.com (Y.T.); erzhan.imanbayev@mail.ru (Y.I.); zhambolova.ainur@mail.ru (A.Z.); aleka4204@mail.ru (A.K.)

**Keywords:** bitumen, modification, petroleum coke, carbon nanomaterials, rheological properties

## Abstract

Carbon-based nanomaterials have emerged as a promising strategy for bitumen modification to enhance the mechanical and thermal performance of pavements. This review evaluates the present advancements in the inclusion of coke and carbon nanomaterials (CNMs) such as carbon nanotubes (CNTs) and graphene into bituminous systems. The findings and limitations of recent experiments in synthesis procedures along with dispersion methods are deeply explored to determine their impact on the rheological properties of bitumen as well as aging resistance and durability characteristics. Petroleum coke enhances bitumen softening points by 10–15 °C and causes up to 30% improvement in rutting resistance while simultaneously prolonging material fatigue life and aging resistance. Bitumen modification through petroleum coke faces challenges in addition to mixing difficulties due to its high viscosity. Moreover, the incorporation of CNTs and graphene as CNMs shows significant enhancements in rutting resistance with improved tensile strength, lower additive requirements, and enhanced dispersion. Both the superior mechanical properties of carbon nanomaterials and processing advancements in nano-enhanced bitumen have the capability to solve technical issues including material costs and specialized mixing processes. Combining coke with CNMs to enhance performance is a future research direction, which could result in economic and scalability considerations. This review comprehensively explores insights into physicochemical interactions, performance outcomes, and processing techniques, crucial for the development of sustainable, high-performance bitumen composites tailored for next-generation infrastructure applications.

## 1. Introduction

Bitumen is known for playing a critical role in road construction due to its viscoelastic nature, durability, and adhesive properties. Traditional bitumen requires modification because it shows several limitations in resistance to temperature changes and aging as well as susceptibility to cracking during repeated mechanical stress. The performance and longevity of pavements is negatively affected by three major aging mechanisms that include bitumen’s thermal instability and cracking together with oxidative aging and ultraviolet degradation combined with susceptibility to moisture [1]. Researchers over the past few decades have conducted extensive investigations into bitumen modification to enhance its structural properties as well as rheological and environmental features through various physical and chemical methods [2]. Carbon-based additives including petroleum coke and carbon nanomaterials (CNMs) have become important research subjects because they show promise for strengthening bitumen’s properties at both mechanical and thermal levels [3,4]. The superior resistance of carbon-based reinforcements at high temperatures with enhanced mechanical properties and extended stability makes them desirable choices for sustainable bitumen modification compared to traditional polymeric additives.

The engineering of bitumen for future asphalt pavements necessitates an in-depth understanding of its chemistry, structure, and rheology. The modification of bitumen using advanced materials aims to address issues related to fatigue resistance, oxidative aging, and moisture susceptibility. Physical modification methods primarily involve the addition of polymers, mineral fillers, and other reinforcements to enhance the bitumen matrix. The improvement in bitumen’s performance results from a combined application of polymers along with mineral fillers and reinforcements that create an enhanced durable matrix [2]. Extensive studies have been conducted on styrene–butadiene–styrene (SBS) and ethylene–vinyl acetate (EVA), while carbonaceous materials such as coke and CNMs show superior sustainability performance [5]. The effective incorporation of coke and CNMs into bitumen systems requires complete solutions to handle processing scalability together with phase separation and dispersion stability challenges. Bitumen emulsions, which have been widely used in road construction, also benefit from the addition of reinforcement materials. Research demonstrates that emulsified bitumen effectively decreases construction expenses while extending pavement duration [6]. New bitumen formulations through modification have enhanced resistance to three major pavement damage types including rutting and thermal and water-induced cracking [7]. The integration of nanomaterials and other advanced fillers is a possible solution to improve bitumen’s mechanical strength while enhancing its cohesion properties [8,9]. The incorporation of polymer waste into bitumen modification systems critically enhances both durability and resistance toward external elements, providing further sustainable solutions for road construction [10].

**Figure 1:**
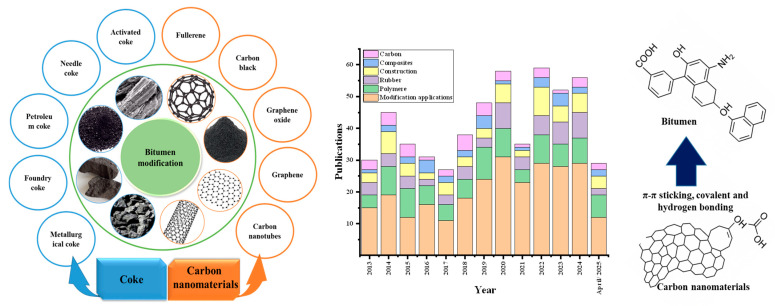
An overview of coke and CNMs used in bitumen modification, their interaction mechanisms with bitumen, and publication trends from 2013 to 2025 (data retrieved from the “Scopus” database on 12 April 2025 using the terms “Bitumen modifications, carbon nanomaterials, coke, carbon nanotubes, and applications” to search within the article title, abstract, and keywords).

This review work mainly aimed to obtain a deep understanding of the integration of coke and CNMs into bitumen by evaluating the present studies. In Figure 1, the types of coke (e.g., needle coke, petroleum coke, and foundry coke) and CNMs (e.g., graphene, graphene oxide, carbon nanotubes) utilized for bitumen modification are illustrated. Figure 1 further illustrates the binding mechanism of CNMs and bitumen through three main bonds (π–π stacking, covalent bonds, and hydrogen bonding) and presents the rising publications trends from 2013 to April of 2025, which demonstrates the growing interest in bitumen modification.

Petroleum coke has become a superior choice for industrial waste management because of increasing construction material standards for low-carbon solutions which reduce the environmental effects of road infrastructure by using waste products. The advancement of alternative materials for bitumen modification might have environmental concerns together with economic advantages. Furthermore, researchers have investigated plastic waste additives to modify road construction materials due to their potential to lower bitumen requirements and improve pavement durability [11]. Scientists have also discovered micro- and nano-dispersion additives which bring further substantial performance enhancements to bitumen by increasing its capabilities to withstand deformation, fatigue, and aging processes [5]. Similarly, waste-material-based bitumen mixture development and polymer-modified bitumen are other sustainable options for enhancing structural integrity and reducing maintenance costs [12,13].

Among these modification materials, coke together with other carbon-based materials is vitally important for developing high-performance bituminous binders [3]. Petroleum coke derived from oil refining operations provides raw materials for multiple CNMs used in bitumen reinforcement like graphene, carbon nanotubes, and carbon black [14]. The addition of CNMs improves bitumen’s durability while saving the environment due to industry waste products like coke being redirected into useful applications. The existing process of road construction continues to pose substantial environmental challenges as a main issue. The environmental aspects of traditional asphalt production also confirm the necessity for sustainable modification solutions to reduce both pollution and energy consumption [15]. Bitumen modified with innovative carbon-based reinforcement elements demonstrates both superior material performance and environmental benefits that reduce construction-related ecological consequences. This review evaluates bitumen modification with coke and carbon nanomaterials to understand their effects on bituminous binder properties related to rheology and mechanics as well as environmental considerations. This review also combines current research results to identify reinforcement processes together with improved production methods for modified bituminous materials to ensure their long-term performance characteristics. Additionally, this review explores the utilization of industrial by-products such as coke for producing advanced asphalt composites according to contemporary sustainable road building methods and how to overcome processing difficulties with carbon nanomaterials within bitumen to create improved bitumen binders from waste materials with nanostructured binders designed for environmentally friendly road development.

## 2. Materials and Methods

This review article was shaped by a systematic, organized bibliographic analysis which aimed to evaluate the recent advancements in the modification of bituminous systems by introducing coke and CNMs. A comprehensive literature search was performed through databases like Web of Science, Scopus, and ScienceDirect to maintain scientific rigor and academic integrity. These platforms provided comprehensive peer-reviewed scientific literature along with chemical engineering and civil engineering content. This study examined publications from 2013 to 2025 with a particular focus on the years 2024 and 2025 to capture the recent experimental developments and advancements in materials. Multiple Boolean combinations of the search terms “bitumen modification”, “coke” along with “carbon nanomaterials”, “carbon nanotubes”, and “graphene”, as well as performance-related terms such as “thermal cracking”, “rheology”, “aging resistance”, and “mechanical performance”, were used to filter the results. This review included peer-reviewed articles from academic journals with strong publication reputations.

This review further included studies which investigated the synthesis processes and blending procedures along with functional assessment methods for bitumen modified with coke, coke derivatives, and CNMs. These studies included (i) coke synthesis methods and modification protocols utilizing coals tar pitch as well as lignite-derived coke and needle coke alongside (ii) characterization evaluations of modified bitumen through conventional analytical procedures such as Fourier transform infrared (FTIR) spectroscopy, X-ray diffraction (XRD), Raman spectroscopy (RS), and scanning and transmission electron microscopy (SEM and TEM). Studies which contained insufficient empirical data and research based solely on theoretical models or unmodified bitumen were excluded because they lacked technical relevance. The focus centered on studies delivering experimental evidence depicting how coke or CNMs bonded with the bitumen matrix and their impact on rheological characteristics, aging stability, thermal properties, and durability.

Each chosen article provided structured data about nanomaterial and coke morphology, synthesis methods, synthesis protocols, and performance measurements of the viscosity, softening point, penetration, complex modulus, and phase angle. The methodology embraced both comprehensive data collection and integrative analysis which established rigorous research standards and built a solid basis for understanding modern bitumen modification.

## 3. Bitumen Modification

**Figure 2:**
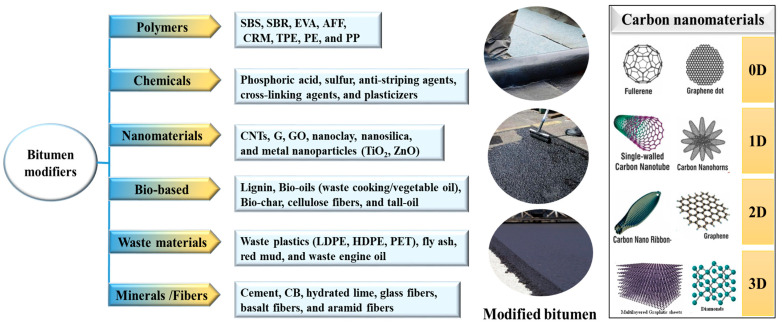
The types of carbon nanomaterials and versatile additives used for the modification of bitumen.

The modification of bitumen stands as a vital process which improves the functionality and longevity of bituminous materials utilized throughout road construction. The modification process includes the integration of different additives which strengthen the resistance to rutting and cracking and improve the aging performance. The modification of bitumen remains essential because it enables performance consistency through diverse environmental and traffic conditions. The modification of bitumen currently takes place through six main strategies, shown in Figure 2: polymer modification, chemical modification, and the incorporation of nano-additives, bio-based nanomaterials, industrial waste materials, and minerals and fibers. Bitumen modified using a combination of the polymers SBS and EVA has the superior elastic properties and temperature tolerance needed for advanced use applications. Ozonation along with functionalized additive additions represent chemical modification approaches that enhance oxidation resistance as well as thermal stability [16]. The replacement of traditional bitumen-modifying materials with bio-based chitosan demonstrates both sustainable practice and property improvement through reduced environmental impacts during modification processes [17].

Traditional modifiers composed of polymers and rubber are widely used to enhance bitumen performance, yet they present several challenges regarding their costs, aging characteristics, and environmental stability [18]. Advanced carbon-based modifiers such as CNTs, graphene, and semi-coke ash enhance bitumen properties and deliver better results. Table 1 displays advanced carbon-based modifiers which outperform conventional materials by significantly enhancing both the mechanical properties and operational lifespan of bitumen, particularly through the usage of CNTs and graphene.

These materials increase the rutting resistance and fatigue life and improve the softening point, demonstrating high value for advanced bituminous solutions. CNTs prove successful in enhancing bitumen’s properties by improving the fatigue resistance and rheological behavior due to their exceptional mechanical strength and thermal properties [20]. The addition of graphene to bitumen results in improved viscosity together with enhanced adhesion properties and rutting resistance, which positions it as a potential material for high-performance asphalt mixtures [23]. Semi-coke ash serves as a low-carbon modification for bitumen due to its ability to enhance thermal stability while decreasing the environmental effects of modification [24]. Current studies about nano-additive modification with specific chemical groups show important enhancements in the mechanical and physical characteristics of bitumen.

A modified bitumen material requires specific performance testing, such as that of its rutting resistance, fatigue resistance, and degradation resistance, to confirm its durability and outdoor functionality. Rutting resistance evaluation stands as a vital performance measure for preventing layer damage under heavy-traffic conditions. Additionally, modified bitumen must exhibit fatigue resistance to withstand repeated loading cycles and prevent premature cracking. Resistance to aging stands as an essential factor because bitumen suffers degradation when exposed to oxidation and ultraviolet (UV) radiation. During its application, modified bitumen should demonstrate both excellent processing capabilities and compatibility with previously utilized asphalt mixtures. Research findings confirm that eco-friendly polyethylene (PE) pyrolytic wax additives provide sustainably enhanced tensile strength as well as aging and deformation resistance to bitumen [19]. Novel carbon-based sustainability-approved modifiers now enable the development of future bitumen products which deliver better performance alongside environmental advantages. The advancement of research in this field will lead to innovative solutions like chitosan-based modification and nano-enhanced additives which will determine how bitumen technology evolves for better infrastructure development with longer-lasting durable and environmentally responsible solutions.

### 3.1. Coke and Coke-Derived Materials

Coke is a carbon-rich solid material obtained primarily through the carbonization of coal or other carbonaceous substances in the absence of air, serving as a critical material in the metallurgical, environmental, and chemical industries. Different types of coke, including metallurgical coke, foundry coke, petroleum coke, and needle coke along with activated coke, alongside their origin, physical characteristics, and usage patterns, are shown in Table 2. Metallurgical coke serves as a blast furnace raw material in ironmaking operations because it maintains 40–50% porosity yet displays 20 to 40 MPa compressive strength together with less than 10% ash content [25]. Foundry coke functions as a primary carbon source for smaller furnaces used in metal melting operations because it offers superior strength capabilities and higher carbon content with larger sizing than metallurgical coke.

Petroleum coke from oil refining yields lower ash matter but shows a sulfur content reaching 6%, therefore requiring desulfurization treatments before further use [26]. Furthermore, needle coke functions as a high-grade manufacturing material for graphite electrodes because its crystal structure maintains low thermal expansion while meeting requirements for hot environments. Activated coke has both environmental and industrial applications because it maintains surface areas beyond 500 m^2^/g, making flue gas purification possible through its outstanding adsorption.

Different synthesis techniques exist for coke production in relation to the desired coke type and its intended application [29]. The production of traditional metallurgical coke requires the high-temperature carbonization of blends from selected coking coals inside by-product coke ovens while maintaining temperatures of between 1000 and 1100 °C for 16–18 h under inert conditions [25]. The carbonization process under this method removes volatile elements to produce a carbon-rich substance with the desired mechanical properties. Petroleum coke forms during refinery delayed coking operations by heating heavy residual oils up to 500 °C, which yields solid coke together with light hydrocarbons [30]. Needle coke production requires feedstock thermolysis combined with heat treatment at temperatures of up to 1400 °C to transform the material from its raw state into finished needle coke [26].

Furthermore, renewable biomass resources undergo pyrolysis or hydrothermal carbonization to create biomass-derived coke through recent technological developments [28]. Among the various methods, high-temperature coal-blending carbonization in by-product ovens is best for the industrial and commercially viable production of metallurgical coke because it optimizes weight yield and material strength performance while ensuring suitable reactivity. Current industrial standard coke production achieves between 70 and 80% weight yield of coke from coal together with valuable by-product recovery including tar, ammonia, and benzene, which enhance the financial results [26]. The primary coke production techniques along with their raw materials and resulting characteristics for various industrial applications are presented in Table 3.

Quality control plays a critical role during this process because different carbon formations of isotropic and anisotropic textures directly affect the chemical strength and reactive properties of the produced coke [32]. The application of variable activation energy models during kinetic studies of coke reduction and melting has given valuable insights into optimizing both the reactions and structural stability performance at high temperatures, which is essential in blast furnace operations [33].

**Figure 3:**
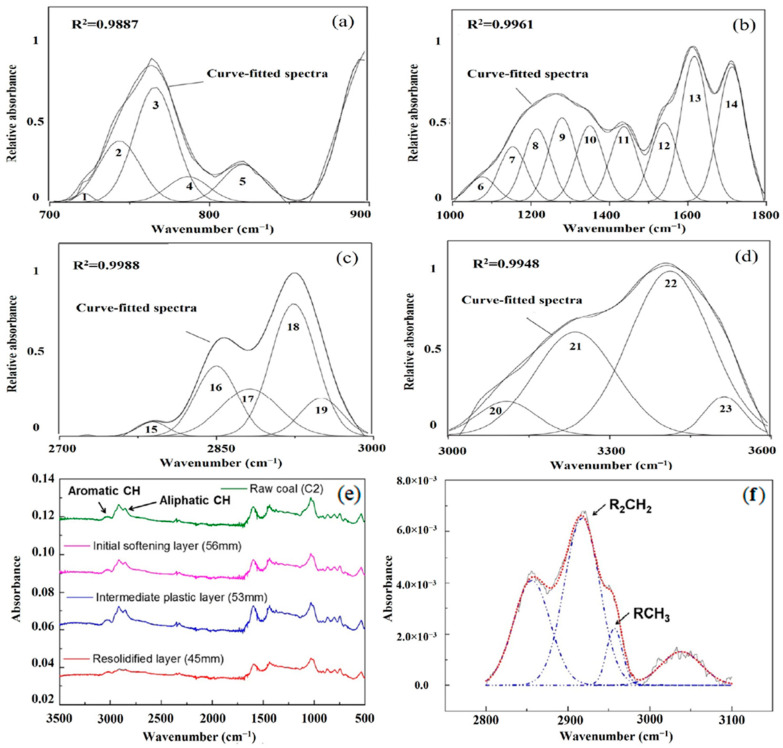
FTIR analysis of raw coal and thermally transformed layers: (**a**) aromatic C–H out-of-plane bending (700–900 cm^−1^); (**b**) aromatic C=C, C–O, and phenolic groups (1000–1800 cm^−1^); (**c**) aliphatic CH_2_ and CH_3_ stretching (2700–3000 cm^−1^); (**d**) broad O–H/N–H stretching (3000–3600 cm^−1^); (**e**) comparison of FTIR spectra across raw, softening, plastic, and resolidified layers showing evolving C–H band intensity, and (**f**) curve-fitted aliphatic CH stretching vibrations highlighting RCH_3_ and R_2_CH_2_ absorption peaks in the 2800–3100 cm^−1^ region. [Adopted from Zheng et al., 2021, [34] under the terms of the Creative Commons CC BY 4.0 license].

Activated coke produced through customized activation methods shows a greater than 90% simultaneous pollutant removal capability for SO_2_ and NO_x_ in environmental applications [27]. The combination of FTIR analysis with XRD, Raman spectroscopy, and SEM tests has provided a complete understanding of how coke and coke-derived porous carbon features are suited for modifying bitumen. Through FTIR analysis, as shown in Figure 3, it becomes possible to monitor the structural changes that petroleum coke undergoes during different thermal softening processes [34]. Two distinct peaks at 754 cm^−1^ and 816 cm^−1^ indicating polyaromatic hydrocarbon clusters from out-of-plane bending vibrations of aromatic C–H bonds are presented in Figure 3a. The aromatic structural units present in petrochemical carbon improve its affinity toward bitumen aromatic zones which helps stabilize the asphaltene fraction [35].

A strong aromatic skeletal vibration at ~1600 cm^−1^ (peak 13) is shown in Figure 3b (1000–1800 cm^−1^) together with C–O and CH_2_ bending vibrations within the 1260–1450 cm^−1^ range. Intercellular bonding with polar groups in oxidized bitumen becomes more effective due to oxygen-containing functionalities found in polymers that enhance interfacial bonding while improving aging resistance [36]. Multiple C-H stretching vibrations can be observed on the spectrum in Figure 3c (2700–3000 cm^−1^). Symmetric and asymmetric CH_2_ stretches appear at 2852 cm^−1^ and 2922 cm^−1^ (peaks 16 and 18), whereas the stretching of CH_3_ occurs at 2955 cm^−1^ (peak 19). The aliphatic chain structures introduced in these modifications improve bitumen softening behaviors through the development of a precise rigidity–flexibility balance.

Figure 3d reveals a broad region from 3100 to 3600 cm^−1^, with the strongest peak 22 at 3392 cm^−1^, which suggests the presence of O–H and N–H vibrations in the sample. Polar functional groups help hydrogen bond with oxidized bitumen, resulting in better bonding between the concrete and the bitumen layer and overall enhanced storage and work performance [37]. Figure 3e also includes the comparative FTIR spectra of raw coal and the different thermal layers (initial softening, plastic, and resolidified). Adjustments in the absorbance intensities of aliphatic and aromatic carbon–hydrogen interactions are due to shifts in the structure of the molecules after heat treatment. Due to this structural change, the asphalt becomes more aromatic, and its carbon domains become more regular, which aids in mixing with bitumen and keeps asphalt from evaporating at high temperatures [38].

Moreover, Figure 3f provides analyzed spectra in the 2800–3000 cm^−1^ range, showing that R_2_CH_2_ and RCH_3_ groups contribute to this part of the spectrum. With this accurate observation of aliphatic stretching modes, we can better understand how coke treatment affects hydrocarbon molecules. However, to fully validate the proposed interfacial interaction mechanisms, further studies using molecular dynamics (MD) simulations, interfacial energy calculations, and thermodynamic assessments (e.g., adsorption enthalpy) are recommended. These tools can complement the spectroscopic results by quantifying interaction strengths at the molecular level and revealing the energetic landscape of coke–bitumen adhesion.

**Figure 4:**
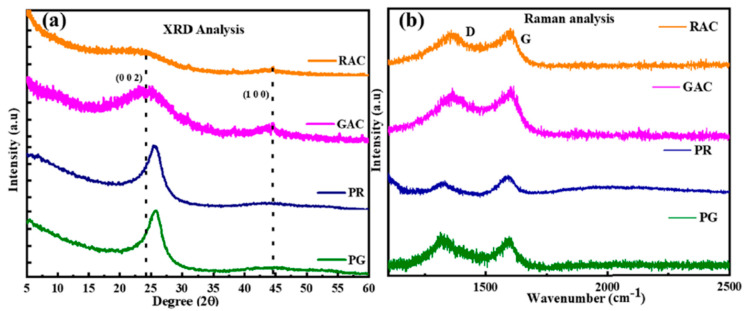
Crystallinity and structural disorder of coke and coke-derived porous carbons: (**a**) XRD patterns of RAC, GAC, PR, and PG showing broad peaks corresponding to the (002) and (100) planes, indicating low graphitization and amorphous structure; (**b**) Raman spectra exhibiting D and G bands for all samples, where increased ID/IG ratios suggest greater defect density and disorder in RAC and PR samples compared to GAC [Reproduced from Benoy et al., 2024, [40] under the terms of the Creative Commons CC BY 3.0 license].

The XRD pattern in Figure 4a shows broad peaks at 2θ values of 25° and 43° that correspond to the (002) and (100) reflectance planes of graphite. When compared to petroleum residue (PR) and petroleum graphite residue (PG) samples, raw activated coke (RAC) and graphitized activated coke (GAC) demonstrate wider and intense peaks, which indicates that these materials exhibit both a partial graphitic character and short-range order structures. Modified bitumen retains better thermal stability and shows stronger resistance to oxidative damage when the structural graphitic features improve [34]. Additionally, the Raman spectra (Figure 4b) exhibit two strong peaks at ~1350 cm^−1^ (D band) and ~1580 cm^−1^ (G band). The combination of structural irregularities with graphitic domains in RAC and GAC produces higher intensity D-band/G-band ratios than PR and PG. The controlled structural disorder of the systems allows for elastic behavior and energy dissipation to enhance the crack resistance during the cyclic tension of bituminous materials [39].

**Figure 5:**
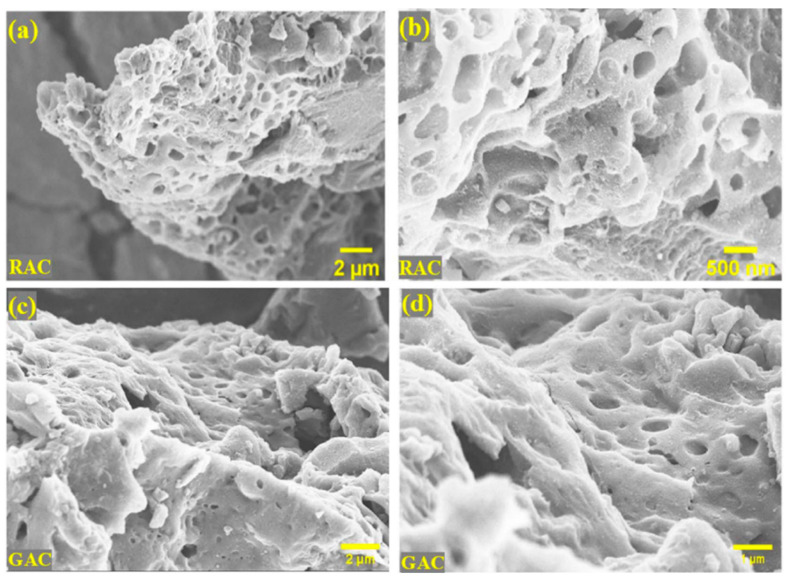
SEM micrographs of porous carbon derived from coke: (**a**,**b**) raw activated coke (RAC) exhibits interconnected macropores and a rugged surface, enhancing adsorption capacity; (**c**,**d**) graphitized activated coke (GAC) reveals denser structures with reduced surface roughness and more stable pore distribution, beneficial for dispersion in bitumen matrices [Adapted from Benoy et al., 2024, [40] under the terms of the Creative Commons CC BY 3.0 license].

SEM micrographs (Figure 5) further corroborate the physical transformation of the carbon material. The raw activated coke (RAC) in Figure 5a,b presents both a large number of open pores and wide pore diameters extending from submicron to several microns. The filler finds adequate attachment to the matrix structure through bitumen penetration because of hierarchical porosity [41]. The structural network of the graphitized activated coke (GAC) in Figure 5c,d indicates denser porosity which remains porous following activation procedures. Studies about the morphology and structure of coke confirm that coke-derived porous carbon can modify bitumen, which can achieve superior thermal conductivity performance along with enhanced fatigue resistance through its special material composition [40]. However, further investigation is needed to establish a quantitative model linking pore distribution with rheological behavior, as well as to define the relationship between the graphitization degree and thermal conductivity to better support predictive performance analysis.

The combination of surface functional groups with hierarchical porosity that exhibits controlled disorder and moderate graphitization produces bitumen modification synergistically to develop advanced rheological and durability-enhanced asphalt products [42]. The compactness behavior of coke particles depends heavily on their particle size distribution as well as porosity since both factors significantly determine the stability of coke used as a filler material in industrial applications [43]. The optimal selection of particle size distributions based on quantitative compaction results produces materials with densities higher than 1.5 g/cm^3^ while enabling enhanced mechanical interlocking.

Coke porosity falls by between 30% and 50% according to production techniques with associated feedstocks, enhancing its performance in construction [44]. The physical characteristics of coke improve cementitious material and coke binding to create uniform concrete microstructures. These materials are optimal for refractory concrete applications due to the exceptional thermal stability of coke, which surpasses 1000 °C in its decomposition temperatures [45]. Additionally, the chemical stability of coke under normal environmental conditions ensures its long-lasting performance when used in construction applications. However, under high-temperature conditions, coke undergoes structural transformations that can modify its reactivity. The carbon purity levels of the material determine its durability and functionality as a reinforcing component in materials where better reinforcing performance occurs at a higher carbon content [46]. The character of coke as a doping agent attracts high levels of interest from researchers due to its effect in optimizing cementitious systems’ properties. The integration of coke into concrete or asphalt increases thermal conductivity along with both the mechanical strength and durability levels [47]. The maximum coke addition at 5% weight leads to a 10% improvement in compressive strength and enhanced thermal resistance in traditional cementitious mixes [48].

The addition of fine coke powders in concrete manufacturing as lightweight aggregate materials reduces concrete density but maintains or improves the compressive strength [48]. The porous profile of coke further enhances concrete moisture retention during curing, which results in improved efficiency and a decreased risk of micro-crack formation. Asphalt pavements heavily benefit from coke-derived additives which enhance the rutting resistance and fatigue life performance at elevated temperatures in road construction projects [49]. Furthermore, the addition of coke to asphalt mixtures results in a 15% improvement in the Marshall stability according to quantitative research [50]. Coke utilization for this purpose simultaneously lowers environmental emissions while maximizing industrial waste products, which supports modern green building standards. The performance of coke in construction applications is affected by quality fluctuations which stem from varying feedstock ingredients and coking methods [51]. Coke serves as a valuable construction material because its unique blend of mechanical attributes and chemical stability and structural versatility enables multiple applications in concrete structures and components as well as roadway surfaces. Studies have revealed potential methods to improve coke properties which open possibilities for creating advanced construction solutions [52].

### 3.2. Mechanism of Coke–Bitumen Interfacial Bonding

The performance of coke-modified binders depends on the bonding of the coke particles with the bitumen, which is the major reason for material homogeneity, stress transfer, and long-term durability. The nature of this interaction includes both physical and chemical mechanisms that depend on surface properties, environmental conditions, and processing parameters. The optimization of these interactions by choosing the right materials, treating the surface, and setting suitable processing conditions is crucial for improving the toughness and mechanical features of coke-modified binders. The main properties of coke which affect both its physical and chemical interactions with bitumen are presented in Table 4, explaining their impact on bonding and its mechanism.

The physical adsorption of coke particles onto bitumen occurs due to van der Waals forces and mechanical entanglement. Because coke particles are porous and rough, bitumen sticks to them more easily and binds better to the rest of the coke [53]. The presence of these morphological features increases the ability of the interface to resist cracks when it is under mechanical stress [54].

Bonding between particles becomes stronger and more durable due to chemical interactions. Coke retains oxygen-bearing functional groups such as hydroxyl, carbonyl, and carboxyl groups, mainly when prepared through controlled oxidation. These surface functionalities can interact with polar molecules within bitumen, mainly asphaltenes and resins, due to hydrogen bonding, π–π interactions, and dipole–dipole attractions [53]. These bonds improve the adhesion and cohesion between phases, even in the presence of thermal and moisture cycles.

Studies on molecular-level interactions confirm that the surface polarity and functionality of both bitumen and solid substrates dramatically affect adhesion. The oxidized surfaces and polar bitumen fractions exhibit stronger bonding behavior compared to their unmodified surfaces according to molecular dynamics (MD) simulations [56,57]. This is similar to coke–bitumen systems, where coke mimics solid mineral behavior due to its carbon-rich structure and surface activity.

The wetting behavior of bitumen toward coke also plays a central role. Better wettability implies stronger interfacial contact, which leads to uniform dispersion and enhanced stress transfer [54]. If the bitumen does not sufficiently wet the coke surface, voids can form at the interface, which may initiate micro-cracks under load or temperature fluctuations [53]. Consequently, using mild oxidation or nanoparticle modification on coke surfaces can be useful.

Environmental stressors such as moisture and freeze–thaw cycles can degrade interfacial integrity. It was observed that interfacial debonding increased during salt–freeze–thaw cycles when the chemical bonding of bitumen and aggregates was not very strong [59]. These findings indicate that strong chemical bonds in the coke particles would help the particles resist moisture damage, especially due to hydrophobicity. Additionally, co-adsorption plays a role in evenly distributing the binder in the aggregate by attracting coke and heavy bitumen parts to the surface, which stabilizes the aggregate’s internal structure and makes it perform better in the long term [58].

### 3.3. Coke as a Modifier for Bitumen

Coke represents a carbonaceous product obtained from coal or petroleum residue which serves critically to modify bitumen for improved results in road construction and waterproofing applications. The capability of coke to modify bitumen relies on the physical characteristics and chemical nature of the specific type of coke, such as petroleum coke, metallurgical coke, and fluid coke, due to their different compositions. The residual product of refined petroleum known as petroleum coke displays characteristics of elevated carbon composition with minimal ash content and excellent thermal resistance that helps strengthen bitumen [60]. Metallurgical coke produced by coal destruction incorporates porous features which enhance its bonding potential with bitumen and improve both the stability and adhesion levels [61]. Fluid coke obtained by delayed coking processes presents both small-scale particles and an expansive surface area that works to change how bitumen reacts to stresses [62].

Coke exhibits multiple physical characteristics and multiple chemical attributes which influence its behavior when interacting with bitumen. The fixed carbon content of petroleum coke exceeds 80–90%, and the volatile matter content remains below 10%, which results in improved material stiffness and strength in bitumen [60]. The porous structure and 60–80% carbon range of metallurgical coke make it further suitable for bitumen interaction [61]. Petroleum coke with its high sulfur content provides bitumen with enhanced resistance to oxidative aging which enhances its long-term durability [63]. The specific surface area of coke particles maintains significant importance because the particles’ size affects how they distribute and interact within bitumen for better mechanical properties [64].

The mechanisms of interaction between coke and bitumen primarily involve adsorption, interfacial bonding, and reinforcement effects. The micro-reinforcing actions of coke particles enhance the stiffness and strength of bitumen while improving load capacity. The porous structure of coke allows bitumen to penetrate its surface, enhancing adhesion and preventing phase separation [55]. The coke surface contains functional groups that consist of polycyclic aromatic hydrocarbons (PAHs) which enable chemical bonding with bitumen while improving its cohesive strength [65]. The incorporation of coke modifies bitumen rheological characteristics by boosting its elastic properties as well as viscosity which leads to superior rutting and thermal cracking performance [66].

The research data presented in Table 5 demonstrates that modified bitumen shows improved performance characteristics according to recent studies. When bitumen is blended with 5–10% petroleum coke, it obtains a softening point increase of 10–15 °C along with a rutting resistance enhancement of 20–30% [62]. The addition of metallurgical coke at levels between 3 and 7% improves fatigue durability by 25–40% and lowers temperature sensitivity, which enables its usage in high-temperature applications [67]. Due to its resistance to oxidative degradation, coke-modified bitumen demonstrates a 30% longer service life compared to traditional bituminous materials [60]. The incorporation of coke into asphalt pavements makes pavements more durable by strengthening their tensile performance while simultaneously protecting against moisture damage [64]. These engineering benefits of using coke in bitumen modification include both increased mechanical strength and improved thermal stability and durability. Engineering applications benefit from customized bitumen properties through the strategic selection of both the coke type and concentration, which results in improved sustainability and performance outcomes in road construction along with industrial applications.

### 3.4. Carbon Nanomaterials (CNMs)

The nanoscale dimensions and unique carbon atom hybridization properties of carbon nanomaterials have led to their classification into the zero-dimensional (0D), one-dimensional (1D), two-dimensional (2D), and three-dimensional (3D) structures presented in Figure 2. Due to their exceptional photoluminescence properties alongside their high surface-to-volume ratio and exceptional biocompatibility, such materials find unique value in biological imaging and sensing applications. Their fluorescence-emission tuning capability and quantum efficiency beyond 50% make CQDs an ideal material for complex optical applications [68].

The characteristics of CNTs result from their cylinder shape with high aspect ratios, which makes them excellent materials for one-dimensional applications. The carbon nanotube family consists of three main types including single-walled (SWCNTs), double-walled (DWCNTs), and multi-walled carbon nanotubes (MWCNTs). SWCNTs display diameters of between 0.4 and 2 nanometers while maintaining exceptional electrical properties and tensile strength, and MWCNTs possess diameters of up to 100 nanometers and superior thermal stability for the mechanical reinforcement of composites [69]. Two-dimensional carbon nanomaterials include graphene together with its derivatives, whose sp^2^-hybridized carbon arrangement creates a hexagonal lattice structure in a single atomic layer. Graphene exhibits exceptional electron mobility (200,000 cm^2^/V·s) along with thermal conductivity of up to 5000 W/m·K together with 130 GPa mechanical strength, which makes it useful in energy storage technology and the electronic and composite fields [69,70]. The properties of these materials make them suitable for supercapacitors and catalyst support systems as well as applications involving oil absorption [71].

The production methods for carbon nanomaterials depend on their specific nature and dimensional structure, using techniques such as arc discharge, laser vaporization, chemical vapor deposition (CVD), and pyrolysis methods (Table 5). The arc discharge synthesis method enables the production of SWCNTs with high crystal quality at up to 90% yield in controlled environments yet produces various nanotube types while demanding extensive separation procedures [72]. Laser ablation produces SWCNTs of high purity alongside narrowed diameter distributions, yet it exhibits high energy usage and restricted scaling capabilities [69]. Among these, CVD is widely recognized as the most efficient and scalable method [73], especially for the synthesis of 1D and 2D carbon nanostructures such as CNTs and graphene. The CVD technique also enables rapid CNT synthesis above 10 μm/min alongside the production of materials with purity exceeding 95% [68].

The construction applications of CNT-reinforced composites enable greater than 200% and up to 300% increased tensile strength together with 25% to 30% reduced material weight, so these composites help achieve sustainability targets [74,75]. The integration of inexpensive carbon black nanocarbons into bituminous materials produces products that demonstrate better elasticity and ultraviolet protection along with thermal resistance while extending road pavement rutting resistance and fatigue life. CVD stands as the most productive and versatile synthesis technique that enables the production of high-quality carbon nanomaterials in all dimensions, thus ensuring their continuing significance for advancing technology sectors [71].

Among the various synthesis methods listed in Table 5, CVD and pyrolysis emerge as the most viable options for producing carbon nanomaterials tailored for bitumen modification. Using this method, high-quality nanostructures such as CNTs and graphene with excellent mechanical and thermal properties can be synthesized, yet it involves high synthesis temperatures and operational costs. Similarly, pyrolysis is a cost-effective and energy-efficient method, particularly suitable for producing carbon black and amorphous carbon, which are known for enhancing elasticity, thermal stability, and UV resistance. Thus, based on cost, material compatibility, and energy considerations, pyrolysis is the most practical choice to produce carbon nanomaterials for large-scale bitumen modification applications. Advanced characterization techniques, including Raman spectroscopy, X-ray diffraction, and electron microscopy, are essential for confirming the structural integrity, dispersion quality, and interaction of CNMs within construction matrices [76,77]. The quality assessment of 2D CNMs including graphene and graphene oxide applied in concrete and asphalt composites depends on tools for optical characterization [78].

**Table 5:** Summary of major synthesis methods for carbon nanomaterials, with synthesis parameters and scalability potential.

No.	Synthesis Method	Raw Materials	Synthesis Temperature (°C)	Proposed Production Materials	Scale Sensitivity	Reference
1	Arc discharge	Graphite electrodes and inert gas (He/Ar)	4500–5500	SWCNTs, MWCNTs, and fullerenes	Lab to medium	[72]
2	Laser ablation	Graphite target, laser beam, and metal catalyst	3000–3500	High-purity SWCNTs	Lab-scale only	[69]
3	Chemical vapor deposition	Hydrocarbons (e.g., CH_4_, C_2_H_2_) and metal catalyst (Fe, Co, Ni)	600–1200	SWCNTs, MWCNTs, and graphene	Industrial scale	[68]
4	Pyrolysis	Organic precursors (e.g., sugars, polymers)	400–900	Carbon dots, amorphous carbon, and carbon black	Medium scale	[68]
5	Hydrothermal/Solvothermal	Biomass, glucose, citric acid, and organic solvents	150–300 (autoclave)	Carbon quantum dots and graphene quantum dots	Lab scale	[68]
6	Template-assisted method	Polymer/oxide template + carbon source	500–800	3D graphene foams and CNT forests	Lab to small	[71]
7	Ball milling	Graphite flakes	Room temp	Amorphous carbon and graphene nanoplatelets	Industrial scale	[69]

The strong mechanical properties, physical capabilities, and chemical properties of carbon nanomaterials, specifically carbon nanotubes, make them powerful modifiers for construction elements such as concrete and bitumen. CNTs possess mechanical properties that make them stand out from other construction materials, with a tensile strength exceeding 63 GPa and a Young’s modulus reaching more than 1 TPa. The addition of CNTs to concrete causes a 25% increase in compressive strength and more than 30% improvement in flexural strength because of their ability to bridge cracks and their strong bond with cementitious matrices [79]. When CNTs receive nitrogen treatment, they show better mechanical properties because the resistance to load transfer and stiffness rise, leading to enhanced application opportunities in structural composites [80]. The performance of the pavements and advanced infrastructure systems depends on the carbon nanomaterials due to their heat and electricity transfer with exceptional efficiency. The high thermal conductivity of CNTs at 3000 W/m·K improves pavement asphalt heat dissipation, reduces rutting during thermal cycles, and extends the product life in hot regions [81]. The conductivity of these materials allows them to function as embedded sensors for concrete and asphalt which detect strain and damage conditions across their structure. The small dimensions of cemented carbon nanotubes enable them to penetrate micro-voids in construction materials, resulting in minimized permeability together with enhanced durability [82].

The inert nature of carbon nanomaterials allows them to bind with host matrices better when combined with the surface functionalization of polar groups. Functionalization methods such as oxidation and silanization create –COOH, –OH, and –NH_2_ functional groups to boost the chemical bonds between cement and polymer–bitumen blends. The direct synthesis of carbon nanomaterials on fillers like aggregates or fibers has been presented as a modern approach which enhances interfacial bonding strengths while cutting down manufacturing steps [83]. Nanomaterial dispersion through this method produces a uniform distribution which enhances both the mechanical performance and thermal properties of the finished products. CNMs act as dopants to modify the structural composition and functional outcomes of polymers and asphalt materials. The addition of CNTs to polymer–bitumen nanocomposites enhances asphalt binder resilience and reduces rutting and fatigue resistance while enhancing elasticity, thereby making them appropriate for road and airfield construction. Polymers mixed with CNTs at concentrations of between 1 and 3 weight percent exhibited a 50% boost in fatigue performance and markedly stronger thermal tolerance [84]. The combination of polypropylene and multi-walled CNTs creates a composite that shows enhanced tensile strength and thermal conductivity properties suitable for road layer reinforcement and geotextile applications in pavement structures [85,86].

The use of nanomaterials in road construction enhances the bonding strength between binders and aggregates while making them less sensitive to moisture effects. Research has shown that the wet approach to adding CNTs to bitumen creates better dispersion while consistently improving performance measures by 60% for the softening point and more than 40% for the Marshall stability [87]. Smart infrastructure applications such as smart pavements and piezoelectric concrete and self-healing asphalt become possible through the multifunctional nature of CNMs [88].

### 3.5. CNMs as a Modifier for Bitumen

**Figure 6:**
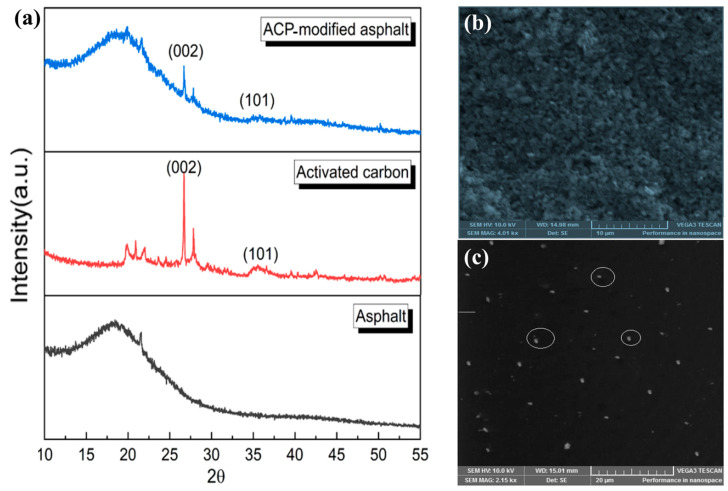
(**a**) XRD patterns of asphalt, activated carbon, and ACP-modified asphalt (reproduced from Wang, Y., 2024, [91] with permission from Elsevier); (**b**) SEM image of porous carbon black; and (**c**) SEM image showing dispersion of carbon black in asphalt matrix (reproduced from Rafi, J., 2018, [92] under Creative commons CC BY 4.0 license).

Carbon nanomaterials, which include carbon nanotubes (CNTs) along with graphene, carbon black, and fullerenes, have received notable interest regarding their use in bitumen modification because of their exceptional thermal, electrical, and mechanical properties [69]. CNTs’ tensile strength of up to 100 GPa and high aspect ratio boost the capacity of bitumen to bear loads. Graphene makes up a single layer of sp^2^-bonded carbon atoms that works as an effective dispersion agent while providing strengthening properties which improve thermal conductivity and oxidation resistance [89]. Carbon black serves as a reinforcing filler to make bitumen stronger and more resistant to aging and ultraviolet light and increases its stiffness and viscosity [90]. Figure 6a presents the XRD patterns of asphalt, activated carbon, and activated carbon powder (ACP)-modified asphalt, offering insight into the structural characteristics and phase composition of each material.

The asphalt sample exhibits a broad diffraction hump centered around 25°, which is characteristic of amorphous carbon, indicating a disordered structure with no significant crystalline domains. Raw bitumen exhibits a typical pattern due to it containing no extended structural organization [76,93]. The activated carbon sample produces well-defined strong peaks at 2θ = ~25° and 43° corresponding to graphitic carbon (002) and (101) planes. X-ray data indicates both improved crystallinity quality and graphitic order in carbon structures following activation processes, which demonstrates the conversion of disordered carbon structures into ordered graphitic domains [94,95]. The dominant (002) peak indicates standard graphitic interlayer distances which reveal the sp^2^-hybridized carbon layers [95]. When activated carbon is added to the asphalt matrix, the resulting ACP-modified asphalt exhibits both original bitumen amorphous features and activated carbon crystalline characteristics. The (002) and (101) peaks present in ACP-modified asphalt show the successful retention of the activated carbon structural order in mixing processes, which results in better material performance [91,96].

The scanning electron microscopy images in Figure 6b,c display the surface features of pure carbon black (Figure 6b) and asphalt with carbon black nanoparticles at a nanometric level (Figure 6c). The carbon black surface in Figure 6b demonstrates amorphous carbon characteristics with numerous pores and rough edges that lead to large surface areas and active sites, which are crucial for binding with the asphalt components. The porous texture serves as a key factor for strong physical adsorption and maintains better mechanical interlocking between bitumen molecules [78]. Observations of carbon black dispersion in the asphalt matrix are shown in Figure 6c. Multiple carbon black particles show well-spaced distribution patterns throughout the asphalt in the circled areas of the image. The dispersion remains stable due to the homogenous carbon black distribution, thus producing improved reinforcement of the bitumen matrix [92]. Such a well-distributed particle arrangement enhances asphalt resistance to deformation while providing protection against fatigue and thermal cracking [97,98].

The structural observations confirm that the modified asphalt binder develops robust bond networks with the modifier, leading to improved stiffness values and enhanced durability properties [99,100], as demonstrated by high-temperature durability tests. The unambiguous change in the surface characteristics through the modification processes verifies that carbon nanomaterials play an active part in strengthening asphalt structures and boosting their extended useful life for pavements [101,102]. The combined structure supports improved interaction at the molecular level, potentially reinforcing the asphalt and enhancing its mechanical behavior and resistance to environmental degradation. Carbon black and other carbonaceous fillers, such as activated carbon, serve as reinforcing agents by increasing the stiffness, viscosity, and aging resistance of bitumen while also protecting it against ultraviolet radiation [90].

**Figure 7:**
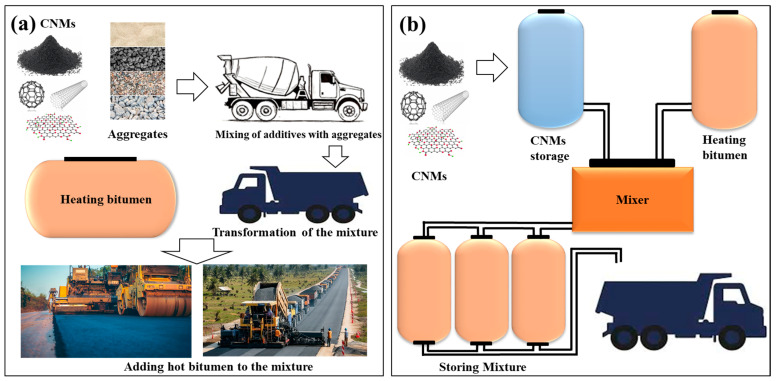
(**a**) The process steps of the dry modification method for the integration of CNMs into bitumen and (**b**) the process steps of the wet modification method for the integration of CNMs into bitumen.

Moreover, the hybrid nature of ACP-modified asphalt, combining amorphous and ordered phases, contributes to its superior viscoelastic properties and energy dissipation ability under mechanical stress, qualities that are highly desirable for extending the lifespan of road pavements [103]. The incorporation of fullerenes, also known as spherical carbon molecules, into bitumen composites leads to the further development of self-healing properties alongside flexibility [104]. These materials enhance bitumen at its molecular level, producing durable and sustainable asphalt pavement structures. The integration of carbon nanomaterials within bitumen utilizes two essential methods, named the dry and wet approaches, presented in Figure 7.

The dry method combines nanomaterials with bitumen through mechanical stirring at specified proportions, involving the mixing of additives with aggregates, mechanical mixing, and the addition of hot bitumen to the premixed aggregate–carbon nanoparticles (Figure 7a). Nanoparticles tend to experience limited distribution within the bitumen matrix when employing this straightforward mixing method, which potentially affects material performance. The wet method incorporates carbon nanomaterials into bitumen by dissolving or dispersing them using solvents before addition, as shown in Figure 7b. The nanomaterials achieve optimal dispersion and better interaction with bitumen when applied through the wet method, and this improved performance enables better modification of material properties [105]. Research indicates that the most effective bitumen modification is achieved through CNT and graphene concentrations ranging from 0.5% to 2% because higher amounts might trigger material property degradation through aggregation [104].

Recent findings show that carbon nanomaterials produce extensive changes in the properties of bitumen, as shown in Table 6. The incorporation of graphene and CNTs enables bitumen to resist degradation beginning at 280 °C and reaching 380–400 °C [104]. Asphalt pavements operating in hot climates benefit from the enhanced thermal resistance which improves their longevity. When 1% graphene is added to bitumen, it increases the softening point by 10–15 °C, which improves resistance to high-temperature deformation [89]. The incorporation of CNTs helps decrease the thermal conductivity loss rate during bitumen aging, thus extending the operational lifespan of asphalt pavements. The combination of CNTs and graphene tends to elevate bitumen viscosity through increased resistance to rutting. The incorporation of 1% CNTs into bitumen resulted in a viscosity boost of 30–40% compared to unmodified bituminous samples while enhancing its ability to withstand heavy traffic loads [106]. High-performance bitumen results from carbon black addition because it enhances flow characteristics that make pavement construction more manageable.

The rutting resistance of road surfaces increases through the inclusion of carbon nanomaterials, enabling better durability during heat extremes. The study conducted by Yang et al. revealed that exposing bitumen to 1% CNTs increased its rutting resistance by between 25 and 35% [105]. CNTs enhance the material’s rigidity and load-bearing capability through reinforcement, which leads to improved rutting resistance. The addition of carbon nanomaterials along with coke improves the performance outcomes of bitumen structures. The combined addition of CNTs and petroleum coke to bitumen to a volume ratio of 30% resulted in a dramatic improvement in fatigue life and moisture damage resistance compared to single additives [64]. The interacting porous structures and CNT/graphene nano-reinforced coke enhance bitumen’s rheological properties, creating durable road-building materials.

The combined system of coke with carbon nanomaterials produces a cooperative strengthening impact that enhances bitumen’s structural performance substantially. Bitumen receives its micro-reinforcement from coke particles with nanomaterials, providing molecular-level strengthening to deliver multi-scale composite properties. The addition of 5–10% coke with 0.3–0.5% CNTs or graphene results in 15–20 °C softening point increases alongside 30% oxidative aging reduction and 40% tensile strength improvement [64,65]. Coke works as a dispersion enhancer which minimizes nanomaterial aggregation and leads to optimal bitumen material distribution [67]. Such synergistic effects specifically benefit high-performance asphalt by delivering an extended lifespan alongside enhanced sustainability.

Both coke and nanomaterial modifications improve bitumen’s properties, but their implementation requires the resolution of specific challenges and limitations to achieve broader adoption. The results in the previous sections confirm that both coke and nanomaterials effectively enhance bitumen’s mechanical and thermal properties. However, their performance varies, and their interaction with bitumen exhibits distinctive outcomes that merit further discussion. The addition of coke, particularly petroleum coke, to bitumen results in an enhanced softening point and increased rutting resistance. Petroleum-coke-modified bitumen attains a 10–15 °C rise in the softening point, which demonstrates coke’s capability to enhance thermal stability [62]. The fatigue life improvements alongside resistance to oxidative aging show the recognized advantages of the higher sulfur content in petroleum coke for extended longevity [59]. The mixing difficulties along with high viscosity and the aggregation properties of coke in bitumen restrict its usability in actual applications. These process ability issues might impede real-life applications, particularly in road construction where reliable mixing is critical.

## 4. Limitations and Future Prospects

Nanoparticles like CNTs, graphene, and carbon black deliver improved properties compared to conventional materials. The incorporation of CNTs with graphene in nanocomposite formulations shows significant improvements across bitumen mechanical properties and simultaneously increases the structural tensile strength and rutting resistance properties [89]. This modification enables lower amounts to be used compared to coke and reduces the viscosity effects. The integration of graphene into bitumen shows rutting resistance enhancements of up to 35%, establishing nanomaterials as promising agents for advanced bitumen modification [104]. The enhanced dispersion properties of nanomaterials in the bitumen matrix enable better uniformity that leads to superior performance consistency in large-scale road paving applications and industrial use. Despite the above advantages, nanomaterial modifications present specific technical challenges. For instance, achieving optimal dispersion requires specialized mixing techniques like sonication or high-shear mixing methods which enhance the processing difficulty and increase expenses [87]. The expense of nanomaterials represents a major obstacle to their extensive utilization since it restricts accessibility to these materials, particularly for developing nations and large-scale commercial utilization. While this cost limitation is widely acknowledged, few studies have provided detailed techno-economic analyses. Hasan et al. emphasized the importance of integrated lifecycle assessments to evaluate the long-term feasibility and sustainability of nanomaterial applications in road networks [107].

Experimental findings show that incorporating CNTs or graphene with coke produces bitumen with combined properties from both modifiers that include improved rutting resistance and enhanced fatigue life, together with better oxidative aging resistance. These findings are illustrated through real-world case studies. Padwal mentioned that using nano-enhanced materials on roads reduces the time and expense of roadwork projects if linear scheduling methods are applied [108]. Similar to this, Hasan et al. highlighted the importance of carrying out lifecycle assessments when using nanomaterials in road construction to promote sustainability and cost-effectiveness [107].

Future investigations need to include the joint effects that emerge when mixing coke with nanomaterials. In addition, nanomaterials enable the effective dispersion of coke particles throughout bitumen matrices, which results in better distribution and stronger mechanical strength. The addition of nanomaterials to coke during modification can address the poor dispersion and higher viscosity issues by achieving the balanced integration of both additives. The analyzed data indicates that scientists need to overcome multiple issues when optimizing synergistic effects, particularly regarding achieving combined compatibility with proper dispersion uniformity. Tomorrow’s bitumen modification strategies may benefit from combining coke with nanomaterials to address the existing limitations, thus generating superior modification outcomes. Future investigations will concentrate on enhancing the interactions between these modifiers to reach their maximum combined outcomes while addressing technical hurdles and economic obstacles.

Moreover, the large-scale adoption of these materials remains limited due to ongoing challenges despite previous evidence of superior properties regarding stiffness, UV resistance, and deformation recovery. The compositional heterogeneity of petroleum and metallurgical coke against bituminous binders remains the primary challenge because petrochemical and metallurgical coke materials differ greatly in structural composition and surface characteristics and impurity levels [109,110]. The distribution of carbon nanomaterials (carbon black with graphene) presents technical challenges to engineers because agglomeration hinders uniform distribution along with performance benefits in asphalt mixture applications [111].

Standard processing procedures along with field performance dataset variations when applied across different climates and loading conditions limit the development of these materials [112]. The widespread application of nanoparticles in production and paving depends on resolving the health risks and environmental concerns generated by their release into the environment.

More research is needed to optimize coke-based modifiers by structurally redesigning them because the goal is to create pore configuration alteration with enhanced surface activity [113]. Hybrid systems of functional nanomaterials with coke demonstrate a potential to create beneficial synergy effects for durability and crack resistance [114]. Surface functionalization advancements through oxidation techniques and polymer grafting methods will enhance material compatibility and prevent agglomeration between particles. Strengthening performance with local specifications could become faster through the application of machine learning and predictive modeling approaches to develop customized modifiers [110,115]. The full-scale application of carbonaceous additives in road construction requires extensive lifecycle testing with cost–benefit analysis and sustainable safety regulations to guarantee their safe deployment in road development [116,117].

## 5. Conclusions

Coke, together with CNMs, strengthens bituminous matrices while enhancing thermal stability and aging resistance, which corrects fundamental bitumen weaknesses related to cracking, oxidative breakdown, and rutting. Petroleum coke and metallurgical coke stand out as coke additives which produce above 30–40% cumulative performance improvement in key parameters including the softening point, fatigue durability, and moisture resistance. The nanoscale reinforcement properties of CNMs such as carbon nanotubes, graphene, and carbon quantum dots enable them to enhance bitumen properties through π–π stacking and hydrogen and covalent bonding interactions to provide improved elasticity and ultraviolet protection alongside extended service duration. This review provides a comprehensive study of bitumen enhancement techniques involving petroleum coke and CNMs along with their synthesis approaches, modification methods, and evaluation outcomes.

Moreover, modern synthesis approaches such as CVD, hydrothermal processing, and ball milling are discussed, and material purity and scalability are stressed as key elements for industrial use. Among these approaches, CVD stands as the most productive method for synthesizing carbon nanomaterials due to the high-quality carbon nanostructures which enable future improvements in bitumen modification technologies. The extensive assessment of modified bitumen through XRD and additional characterization tools including FTIR, RS, SEM, and TEM demonstrates that precise structural and morphological management leads to optimized performance results.

Further studies are required to address the challenges of maintaining stable dispersion during production and preserving phase homogeneity during processing, together with developing sustainable raw material sources which replace fossil fuel-based materials. The addition of coke with carbon nanomaterials to modify bitumen provides opportunities for creating durable, sustainable bituminous products which could advance future road infrastructure and industrial application durability.

## Figures and Tables

**Table 1:** A comparative analysis of conventional and advanced carbon-based bitumen modifiers based on performance parameters.

No	Base Matrix and Modifier	Modifier (%wt)	Viscosity (Pa·s)	Softening Point (°C)	Rutting Resistance (kPa)	Test Conducted	ASTM Standards (Assumed)	Reference
1	C320 bitumen with SBS polymer	7	At 135 °C, from 0.45 to 0.33	Increased from 48 to 55.2	Increased to 1.32 (27%)	Penetration, softening point, viscosity, stiffness, FTIR, and fluorescence microscopy	D4402, D36, and D7175	[19]
2	80/100 bitumen with NR	3–15	Increased viscosity	Increased from 42 to 55	1.0 to 2.2	Penetration, softening point, and ductility	D36 (softening point)	[18]
3	60/70 bitumen with NC from coconut shell ash	1.5–7.5	Increased viscosity	Increased from 48 to 56 (12%)	1.1 to 2.5	Penetration, softening point, viscosity, ductility, Dynamic Shear Rheometer (DSR), and Rolling Thin-Film Oven (RTFO)	D4402 and D36	[20]
4	Petroleum coke-modified bitumen	5–10	Increased viscosity	52 to 58	Penetration Index (0.1 mm) Form 38–55		D36 and D5	[21,22]
5	Metallurgical coke-modified bitumen	3	Increased viscosity	48	Penetration Index (0.1 mm) 97.9 → 110		D36 and D5	[21,22]
6	Epoxy resin with CNTs	1.7	This study focused on the mechanical properties of the epoxy resin, achieving a tensile modulus of 5.8 GPa and a flexural modulus of 6.0 GPa			Tensile and flexural properties and electrical and thermal conductivity	D638, D790, D257, and E1952	[23]
7	PG 67-22 asphalt with GO	2	The study noted a 39% reduction in creep compliance (J_nr) and a 297% increase in percent recovery (εR), indicating enhanced rutting resistance			Rotational Viscosity (RV), Dynamic Shear Rheometer (DSR), Multiple Stress Creep and Recovery (MSCR), and aging property measurements		[24]

**Table 2:** Different types of coke based on their sources and their practical applications.

No	Type of Coke	Source	Major Properties	Application Area	Reference
1	Metallurgical coke	Coking coal blends	High porosity (40–50%), compressive strength (20–40 MPa), and low ash (<10%)	Blast furnace ironmaking and construction-grade concrete additives	[25]
2	Foundry coke	Coking coal	Larger size, high mechanical strength, and high carbon content	Metal melting in foundries and wear-resistant material fabrication	[26]
3	Petroleum coke	Oil refinery residues	Low ash, high sulfur (up to 6%), and dense structure	Anode production and carbon composites in construction	
4	Needle coke	Aromatic heavy oils	Highly crystalline with low thermal expansion	Graphite electrodes and carbon fiber reinforcements in mechanical parts	
5	Activated coke	Coal and biomass	High surface area (>500 m^2^/g) and microporous structure	Flue gas treatment and reinforced fillers in construction materials	[27]
6	Biomass-derived coke	Biomass (e.g., pyrolysis oil)	Tunable structure and renewable origin	Metallurgical coke substitute and eco-friendly construction composites	[28]

**Table 3:** The role of various coke synthesis methods, highlighting properties and targeted applications.

No.	Synthesis Method	Raw Materials	Properties	Process Temperature, °C	Application Area	Reference
1	High-temperature carbonization	Coking coal blends	High mechanical strength and porosity	1000–1100	Metallurgy and concrete additives for construction	[25]
2	Delayed coking	Petroleum residues	Low ash and high sulfur	480–500	Electrodes and carbon composites for mechanical structures	[26]
3	Needle coke production	Aromatic feedstocks	Highly crystalline and low expansion	Up to 1400	High-strength graphite electrodes and carbon fiber for mechanical components	
4	Biomass pyrolysis	Biomass materials	Renewable and tunable pore structure	~400–600	Eco-friendly coke for metallurgy and green construction composites	[28]
5	Coke deposition on activation	Carbonaceous materials	Narrow pore size distribution	800–1000	Carbon molecular sieves and porous fillers for construction	[31]
6	Activated coke preparation	Coal and biomass	High surface area and adsorption ability	700–900	Flue gas pollutant removal and structural fillers for mechanical applications	[27]

**Table 4:** Physicochemical properties of coke and their influence on interfacial bonding behavior with bitumen, including mechanical, chemical, and environmental interaction effects.

No	Property	Description	Effect on Bitumen Interaction	Reference
1	Surface roughness and porosity	Irregular, porous structure with micro-cracks and cavities	Enhances mechanical interlocking and physical anchoring	[53,54]
2	Surface functional groups	Contains oxygenated groups (–OH, –COOH, –C=O) on surface	Promotes hydrogen bonding and dipole interactions with polar bitumen fractions	[55,56]
3	Hydrophobicity/wettability	Naturally hydrophobic unless oxidized	Influences bitumen spreading and wetting; low wettability reduces interfacial adhesion	[54]
4	Specific surface area (SSA)	High SSA in fine coke powders	Increases contact points for adsorption and interaction with bitumen molecules	[57,58]
5	Thermal stability	Remains stable at bitumen mixing temperatures (140–180 °C)	Allows sustained interfacial bonding during mixing and compaction	[53]
6	Resistance to moisture-induced damage	Low water affinity in unmodified coke	Reduces risk of stripping and debonding under wet or freeze–thaw conditions	[55,59]
7	Co-adsorption behavior	Tends to adsorb to bitumen’s asphaltenes and resins	Enhances binder homogeneity and emulsion stability	[58]
8	Particle size distribution	Variable, dependent on grinding or processing methods	Affects dispersion in bitumen and interface area available for bonding	[54]

**Table 6:** Comparison of properties of bitumen modified with different carbon nanomaterials.

No	Property	Conventional Bitumen	CNT-Modified Bitumen (0.5–2%wt)	Graphene-Modified Bitumen (1–2%wt)	Carbon Black-Modified Bitumen (5–10%wt)	Fullerene-Modified Bitumen (0.5–1%wt)	Reference
1	Softening point (°C)	46–52	55–60 (+9–14 °C)	55–63 (+9–17 °C)	50–57 (+4–11 °C)	48–56 (+2–10 °C)	[89,104]
2	Rutting resistance (% improvement)	–	20–35%	25–40%	15–30%	10–20%	[89,106]
3	Fatigue life (% improvement)	–	15–30%	20–35%	10–20%	5–15%	
4	Oxidative aging resistance (% reduction in aging rate)	–	20–30%	25–35%	15–25%	10–20%	[90,104]
5	Tensile strength (MPa)	0.8–1.2	1.1–1.5 (+20–30%)	1.2–1.6 (+25–35%)	1.0–1.3 (+15–25%)	0.9–1.3 (+10–20%)	[89,106]
6	Moisture damage resistance (% improvement)	–	15–25%	20–30%	10–20%	5–15%	[64,104]

## Data Availability

The data that support the findings of this study are included within this manuscript.
